# Association between nutritional status and mental health among elderly in community health screenings: a cross sectional study

**DOI:** 10.1186/s12877-025-06394-7

**Published:** 2025-09-29

**Authors:** Nurliana Abd Nassir, Siti Sara Yaacob, Noor Azleen Ahmad Tarmizi, Khairatul Nainey Kamaruddin, Nur Amirah Shibraumalisi

**Affiliations:** 1 Department of Primary Care Medicine, Faculty of Medicine, Universiti Teknologi MARA, Sungai Buloh, Selangor, 47000 Malaysia; 2 Department of Public Health Medicine (PHM), Faculty of Medicine, Hospital Al-Sultan Abdullah, Universiti Teknologi MARA, Bandar Puncak Alam, Selangor Darul Ehsan, 42300 Malaysia; 3 Department of Internal Medicine (Geriatric), Faculty of Medicine, Hospital Al-Sultan Abdullah, Universiti Teknologi MARA, Bandar Puncak Alam, Selangor Darul Ehsan, 42300 Malaysia

**Keywords:** Aging, Mental health, Nutritional status, Depression, Anxiety, Elderly, Malaysia, Community screening

## Abstract

**Background:**

Malaysia’s elderly population is projected to reach 15% by 2030. Nutritional status significantly impacts elderly health, with malnutrition linked to depression, anxiety and stress. This study assessed mental health problems and their associations with nutritional status among elderly individuals in Malaysia attending community health screenings.

**Method:**

A cross-sectional study was conducted among elderly participants selected through convenience sampling method from four community health screenings in Kuala Selangor District (June–October 2024). Sociodemographic details, nutritional status (Mini Nutritional Assessment-Short Form, MNA^®^-SF), and mental health problems (Depression, Anxiety, and Stress Scale-21, DASS-21) were obtained. Screening tools were used to assess nutritional and mental health status. Multiple logistic regression was used to examine the associations between nutritional status and mental health problems.

**Results:**

Among 361 elderly participants, the prevalence rates of depression, anxiety, and stress were 12.7%, 9.4%, and 4.2%, respectively. At risk of malnutrition significantly increased the odds of depression (AOR = 10.94, 95% CI = 4.35–27.52), anxiety (AOR = 11.16, 95% CI = 4.10–30.41), and stress (AOR = 5.03, 95% CI = 1.55–16.34), whereas malnutrition increased the odds of depression (AOR = 45.61, 95% CI = 13.20–157.61) and anxiety (AOR = 17.80, 95% CI = 4.93–64.32), although confidence intervals were wide. Female sex and less social engagement were associated with depression. Being a smoker or ex-smoker and having less physical activity were associated with anxiety. Finally, retirees and having sleeping issues were associated with both depression and anxiety.

**Conclusion:**

Nutritional status is strongly associated with of depression, anxiety, and stress in elderly individuals. Early identification, further assessment, and targeted interventions are crucial for improving mental well-being in this population.

## Introduction

The global population is aging, with the proportion of people aged 60 years and above expected to increase from 12% in 2020 to 22% by 2050. In Malaysia, older population is projected to reach 15% by 2030 [1, 2]. As aging increases the risk of chronic diseases, disabilities, and cognitive decline, healthcare providers must remain vigilant and well informed to address the evolving needs of this population.

Beyond physical health challenges, mental health problems, particularly depression and anxiety, are also highly prevalent among older adults and pose a major public health concern [3]. Depression is one of the most common psychiatric disorders in older adults and is characterized by persistent low mood, loss of interest, and functional impairment, whereas anxiety disorders, characterized by excessive worry and heightened stress responses, are also common in older individuals.

A World Health Organization study estimated that 7% of the global older adult population experiences depression, whereas 3.8% experience anxiety, contributing to 6.6% of all disabilities measured in disability-adjusted life years (DALYs) [4]. Locally, the National Health Morbidity Survey 2018 in Malaysia reported a depression prevalence of 11% among elderly individuals [5]. Other studies have shown wide variations, with depression prevalence ranging from 4 to 28%, anxiety prevalence at 22.6%, and stress prevalence at 8.7% among elderly individuals [6–8].

Elderly individuals are at increased risk of mental health problems due to social isolation, physical illness, and cognitive decline [6, 9]. In addition, older individuals with underlying comorbidities, mobility issues, and impaired functional ability are more likely to experience depression [6, 10].

More importantly, increasing evidence suggests that nutritional status plays a crucial role in mental health among older individuals [11–14]. The European Society of Clinical Nutrition and Metabolism (ESPEN) has defined malnutrition as “a state resulting from a lack of intake or uptake of nutrition that leads to altered body composition (decreased fat-free mass) and body cell mass, leading to diminished physical and mental function and impaired clinical outcome from disease” [15].

Malnutrition has been shown to negatively impact cognitive function, mood regulation, and overall mental well-being. It is associated with increased psychological distress, depression, and anxiety in older individuals [12, 16]. Emerging research also points to the potential role of the gut–brain axis, a bidirectional communication system between the gut and brain, in mediating the effects of nutrition on mental health, although the mechanisms remain under active investigation [11, 17]. Given these associations, assessing nutritional status is critical in understanding the mental health burden among older populations.

However, nutritional status is often overlooked in older adults care as one of the associated factors for mental health problems. Most available studies of mental health disorders in elderly individuals have focused more on sociodemographic factors, physical disability, and comorbidities, with limited attention given to nutrition as a contributing factor. The literature review has broadly examined malnutrition as a consequence of mental health disorders rather than exploring malnutrition as a risk factor. Even though depression screening is commonly performed, anxiety and stress remain primarily overlooked in this population. Stress was included in the assessment because, although often considered transient, persistent stress can exacerbate chronic conditions and impair coping mechanisms in older adults. Therefore, evaluating stress alongside depression and anxiety provides a more comprehensive understanding of mental health in this population.

To address these gaps, this study aims to investigate the association between nutritional status and mental health outcomes, specifically depression, anxiety, and stress among elderly individuals in a community setting. Findings may enhance early detection, facilitate further diagnostic evaluation, and support targeted interventions to improve older adults well-being.

## Materials and methods

### Study design and population

This cross-sectional study was conducted among elderly individuals attending four community health screenings in Kuala Selangor District from June 29 to October 19, 2024. These screenings, aimed at general health assessments, primarily targeted the older adults population. The inclusion criteria required participants to be aged 60 years and above with the ability to read and understand Malay or English, whereas the exclusion criteria were those with acute severe illness, clinically diagnosed dementia, or cognitive impairments affecting response reliability.

### Sample size calculation

We used OpenEpi Version 3.01 to determine the sample size on the basis of all the study objectives. The largest sample size estimate was derived from the 27.8% prevalence of depression among older adults in Malaysia reported by Abd Manaf et al. [7], with a 95% confidence level and a 5% margin of error; the calculated sample size was 301 participants. To account for potential nonresponses, this percentage was increased by 20%, resulting in a final sample size of 361 participants.

### Study variables

The dependent variables were mental health problems, namely, depression, anxiety, and stress, which were assessed via the Depression, Anxiety, and Stress Scale-21 (DASS-21) screening tool. The DASS-21 consists of 21 items divided across three subscales with seven questions each for depression, anxiety, and stress. Each item was rated on a 4-point Likert scale reflecting symptom frequency over the past week (0 “Did not apply at all” to 3 “Applied very much or most of the time”). In accordance with the DASS-21 scoring guidelines, subscale scores were summed and then multiplied by two to obtain final scores for depression, anxiety, and stress, respectively. These scores were initially classified into five severity levels (normal, mild, moderate, severe, and extremely severe) based on established cutoff points [18]. For the purpose of regression analysis, and to facilitate clearer interpretation in the context of screening rather than diagnostic assessment, each subscale was further dichotomized into two categories: “absent” (scores in the normal range) and “present” (scores in the mild, moderate, severe, or extremely severe range). Although this approach reduces the granularity of severity levels, it aligns with the study’s primary aim of identifying factors associated with the presence of any mental health problems rather than differentiating between degrees of severity. The cutoff scores used to define the presence of mental health problems were > 9 for depression, > 7 for anxiety, and > 14 for stress after score multiplication [7] [19].

Nutritional status was assessed as the primary independent variable in this study via the Mini Nutritional Assessment-Short Form (MNA^®^-SF) screening tool. The questionnaire comprises six items and each item was scored with a maximum possible total of 14 points, where higher scores indicated better nutritional status, whereas lower scores reflected poorer nutritional status. The scoring categories were as follows: 12–14 signified normal nutritional status, 8–11 indicated risk of malnutrition, and 0–7 classified participants as malnutrition [20].

Sociodemographic variables included age, which was analysed both as a continuous variable and as categorized groups (60–74, 75–84, and ≥ 85 years [21]), gender (male or female), ethnicity (Malay, Chinese, Indian, others), marital status (single, married, widowed, divorced), and living arrangements (alone, with spouse only, with spouse and children, or with extended family) were also recorded. Additionally, household income was categorized as < RM1500 or ≥ RM1500, in accordance with the Malaysian minimum wage guidelines, which are commonly used as a national benchmark for income sufficiency in socioeconomic research, whereas financial dependency was determined by whether participants were independent or relied on children’s allowances or welfare. Education level was categorized as no formal education or primary, secondary, or tertiary education, whereas employment status was recorded as either employed, unemployed or pensioner.

To better understand participants’ demographics in relation to lifestyle, self-reported behaviours were assessed. However, as these were not the primary focus of the study, data were obtained solely through self-reports without the use of specific screening tools. Physical activity was assessed by asking whether the participants engaged in activities such as aerobic exercise, running, brisk walking, cycling, gardening, or swimming, and their responses were categorized as frequently (5–7 times per week), sometimes (2–4 times per week), seldom (once per week), or no activity. Similarly, social engagement was evaluated by inquiring about participation in community activities, including gatherings, community programs, or religious events, with responses classified using the same frequency scale. In addition, sleep problems were assessed by asking participants whether they experienced difficulty falling asleep or maintaining sleep, with responses recorded as either yes or no. Meanwhile, functional independence was assessed on the basis of the ability to perform daily activities, including eating, dressing, bathing, toileting, transferring, and walking, without assistance.

The assessment of clinical characteristics included the presence of comorbidities, particularly cardiovascular conditions such as diabetes mellitus, hypertension, and dyslipidaemia. Additionally, body mass index (BMI) was calculated as weight (kg) divided by height squared (m²) and classified into six categories: underweight (< 18.5 kg/m²), normal weight (18.5–22.9 kg/m²), pre obese (23.0–27.4 kg/m²), obese I (27.5–32.4 kg/m²), obese II (32.5–37.4 kg/m²), and obese III (≥ 37.5 kg/m²).

### Sampling method and data collection

The participants were invited through convenience sampling from elderly individuals attending four community health screenings in Kuala Selangor. With a population of 281,711, this district is the second-largest in Selangor and among the top three with the highest elderly population density [22]. Given this demographic advantage, Kuala Selangor was chosen as the study location. Kuala Selangor was chosen not only because of its high elderly population density but also because of its established collaboration with local leaders, nongovernmental organisations, and healthcare providers, facilitating high participation rates. However, a key limitation of this study is that the participants were self-selected and were attending voluntarily, which may introduce selection bias. Those who participate in community screenings are generally more health conscious, socially engaged, and proactive in seeking medical care. As a result, this study may have underrepresented elderly individuals who are less likely to attend such events, many of whom may have poorer health, higher rates of malnutrition, and unrecognized mental health issues. This selection bias may limit the generalizability of our findings to the broader elderly population, particularly among those who are less socially engaged or less proactive in seeking healthcare.

Community health screening programs are conducted annually by local leaders and healthcare clinics, which are specifically designed for the local community, with a focus on elderly participants and offer various activities, educational sessions, and free health screenings. Our research team set up our own booth for the purpose of data collection. Upon invitation to the booth, participants received an explanation of the study and provided informed consent before proceeding with the four sections of data collection. These included self-administered structured questionnaires and anthropometric measurements conducted by trained researchers. To ensure consistency and reliability, researchers have trained, including demonstrations and supervised practices, on anthropometric measurements and questionnaire structure. Training also included calibration exercises to standardize data collection procedures across sites, with periodic supervision by senior researchers during the study period. The first section captured demographic and clinical characteristics, whereas the second screened mental health problems via the DASS-21. In the third section, weight and height measurements were taken to calculate BMI, followed by the fourth section for nutritional status assessment via the MNA^®^-SF. For each section, different researchers were assigned to guide participants as needed and to explain the results of the screening. Anonymity was maintained throughout the process, and participants with abnormal DASS-21 or MNA^®^-SF screening results were referred to a primary care physician for further assessment to confirm a diagnosis and initiate appropriate management, subject to their consent.

### Study instruments

The MNA^®^-SF in the Malay and English languages was used to assess nutritional status. It is a widely validated screening tool for assessing nutritional status among elderly individuals, demonstrating strong validity and reliability, with reported high sensitivity (97.9%), specificity (100%), and diagnostic accuracy (98.7%) in the Malaysian population [23].

For the mental health assessment, our study used the DASS-21. It is a self-report screening tool that is also available in both the Malay and English languages and allows individuals to assess their depression, anxiety, and stress levels on the basis of their recent experiences. The Malay version of the DASS-21 has demonstrated good internal consistency and construct validity in Malaysian elderly populations, making it a reliable tool for screening mental health conditions. Its validity and reliability were established through confirmatory factor analysis, with satisfactory factor loadings above 0.4 and strong Cronbach’s alpha values for the subscales: 0.84 for depression, 0.74 for anxiety, and 0.79 for stress [24].

While the DASS-21 is a widely used screening instrument for detecting symptoms of depression, anxiety, and stress, it has several limitations. As a self-report measure, it does not provide a clinical diagnosis but serves as a tool to identify individuals at risk of mental health issues on the basis of symptom severity. Given its role as a screening tool, the results obtained from this study may help guide further clinical evaluation to confirm symptom severity and inform appropriate mental health interventions. Its practicality and ease of use make it a feasible option for large-scale screening in elderly populations, particularly in these community health programs, where early identification can facilitate timely support and intervention.

### Statistical analysis

All the statistical analyses were conducted via IBM Statistical Package for the Social Sciences (SPSS) version 29.0. For descriptive statistics, categorical variables are summarized as frequencies and percentages, whereas continuous variables are presented as the means and standard deviations. To identify factors associated with mental health problems, including nutritional status, separate multivariable logistic regression models were constructed for depression, anxiety and stress using the backwards likelihood ratio method. All the models were carefully checked for multicollinearity using variance inflation factors (VIFs), with all VIF values below 2 indicating no significant multicollinearity, and potential two-way interaction terms were evaluated alongside the main effect models. Model fit was assessed using the HosmerLemeshow goodnessoffit test, with all models demonstrating adequate fit (*p* > 0.05). The final multivariable models included only significant predictors of depression, anxiety, and stress. The results are reported as adjusted odds ratios (AORs) with 95% confidence intervals, and statistical significance was set at *p* < 0.05.

## Results

**Fig. 1:**
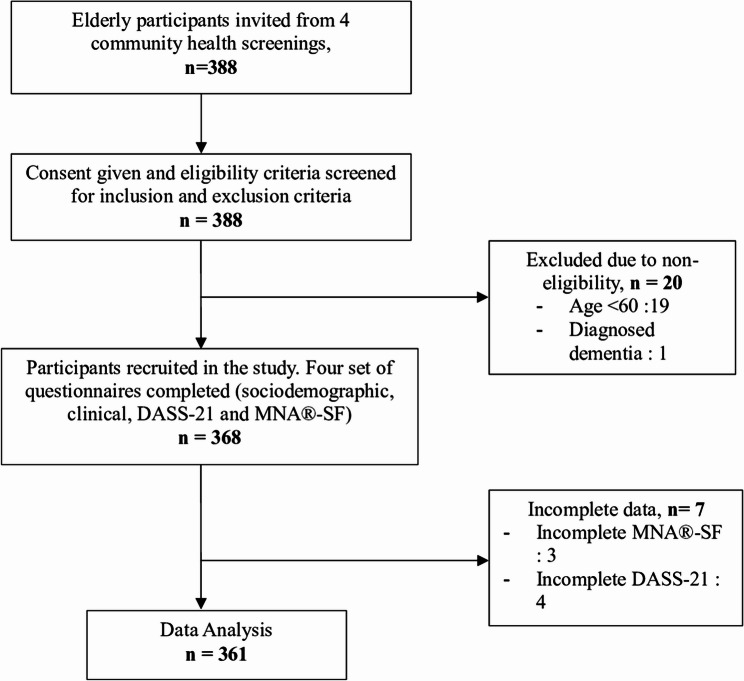
Conduct of the study flow. MNAⓇ-SF, mini nutritional assessment short form; DASS-21, depression, anxiety and stress scale -21

A total of 388 elderly individuals were invited to participate. However, 27 were excluded, leaving 361 participants for the final analysis. The flow of the study is illustrated in Fig. 1.

Among the participants, 59.6% were female, with a mean age of 67.17 years (SD ± 5.74). The majority were aged 60–74 years (86.1%), Malay ethnicity (89.2%), and married (71.5%), while 45.2% had secondary education. Socioeconomically, 57.6% had a household income below RM 1500, and nearly half (48.5%) were unemployed, including 33.0% pensioners. Most lived with a spouse (36.6%) or extended family (20.2%). Furthermore, 78.4% had at least one comorbidity, with dyslipidaemia (58.4%), hypertension (54.6%), and diabetes (35.7%) being the most common. The mean BMI was 27.12 (SD = 5.53), with most patients being overweight (33.2%) or obese (29.1%). Nonsmoking (78.4%) and alcohol abstinence (97.2%) were prevalent, while 28.3% reported sleep difficulties. For lifestyle, 61.2% exercised at least twice a week, and 59.5% participated in social activities at the same frequency. Despite these factors, 95.8% of the participants remained independent in daily activities (Table 1).

**Table 1.** Demographic, socioeconomic, clinical and lifestyle characteristics of the elderly respondents. (N=361)

Variables	Frequency, n (%)	Mean (SD)
Gender		
Male	146 (40.4)	67.17 ± 5.74
Female	215 (59.6)	
Age		
60 to 74 (young-old)	311 (86.1)	
75 to 84 (middle-old)	48 (13.3)	
85 and above (old-old)	2 (0.6)	
Ethnicity		
Malay	322 (89.2)	
Chinese	33 (9.1)	
Indian	6 (1.7)	
Marital status		
Single	7 (1.9)	
Married	258 (71.5)	
Widowed	80 (22.2)	
Divorced	16 (4.4)	
Level of Education		
No Formal Education	41 (11.4)	
Primary: Standard 1 to 6	104 (28.8)	
Secondary: Form 1 to 5 Tertiary: Form	163 (45.2)	
6/College/University	53 (14.7)	
Living arrangement		
Living alone	46 (12.7)	
Living with spouse only	132 (36.6)	
Living with spouse and children	110 (30.5)	
Living with extended family	73 (20.2)	
Job category		
Employed	67 (18.6)	
Unemployed	175 (48.5)	
Pensioner	119 (33.0)	
Income category		
More than RM 1500	153 (42.2)	
Less than RM 1500	208 (57.6)	
Source of Income		
Independent	190 (52.6)	
Dependent	171 (47.4)	
Body Mass Index		
underweight: < 18.5	18 (5.0)	27.12 ± 5.53
Normal: 18.5 – 22.9	62 (17.2)	
Overweight: 23 – 27.4	120 (33.2)	
Obese 1: 27.5 – 32.4	105 (29.1)	
Obese 2: 32.5 – 37.4	41 (11.4)	
Obese 3: >/= 37.5	15 (4.2)	
Comorbidities		
Yes	283 (78.4)	
No	78 (21.6)	
Diabetes	129 (35.7)	
Hypertension	197 (54.6)	
Dyslipidemia	211 (58.4)	
Smoking Status		
Smoker	49 (8.0)	
Ex smoker	29 (13.6)	
Non-Smoker	283 (78.4)	
Alcohol consumption status		
Ever consumed (past or present)	10 (2.8)	
Never consumed	351 (97.2)	
Sleeping problems		
Yes	102 (28.3)	
No	259 (71.7)	
Physical Activity in a week		
Frequent (5-7 times per week)	90 (30.7)	
Sometimes (2-4 times per week)	110 (30.5)	
Seldom (1 time per week)	84 (23.3)	
No activity	56 (15.5)	
Social activity in a week		
Frequent (5-7 times per week)	90 (24.9)	
Sometimes (2-4 times per week)	125 (34.6)	
Seldom (1 time per week)	99 (27.4)	
No activity	47 (13.0)	
Activity of Daily Living (ADL)		
Independent	346 (95.8)	
Dependent	15 (4.2)	

For the prevalence of malnutrition and at risk of malnutrition, of the 361 elderly participants assessed via the MNA^®^-SF, a substantial proportion (23.0%) were identified as being at risk of malnutrition (95% CI: 18.7–27.7%), whereas 6.4% were classified as having malnutrition (95% CI: 4.1–9.4%).

**Fig. 2:**
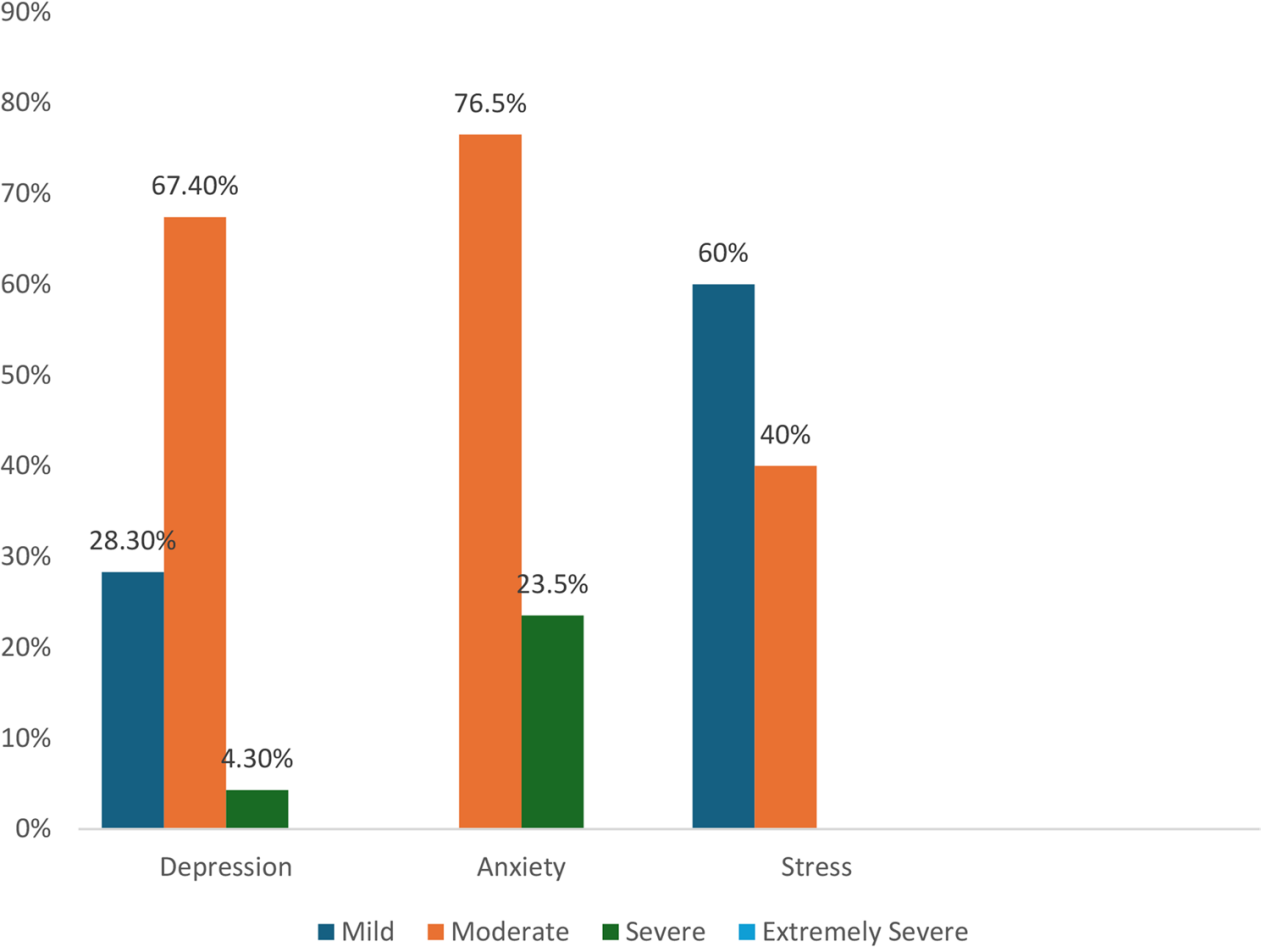
Graph showing the distribution of severity levels for depression, anxiety, and stress among elderly respondents in terms of depression, anxiety and stress scale-21 (DASS-21) scores

The mental health assessment indicated that among the 361 elderly respondents, 12.7% (*n* = 46) experienced depression, with 28.3% classified as mild, 67.4% as moderate, 4.3% as severe, and none as extremely severe. Anxiety was present in 9.4% (*n* = 34) of the respondents, with 76.5% categorized as moderate, 23.5% as severe, and none as mild or extremely severe. Stress was the least common stress experienced by 4.2% (*n* = 15) of the participants, with 60% classified as mild and 40% as moderate, whereas no cases were recorded as severe or extremely severe. Figure 2 show the proportions of elderly individuals who experienced depression, anxiety and stress and their severity according to the DASS-21 score.

Multiple logistic regression revealed that nutritional status was significantly associated with depression, with those at risk of malnutrition having greater odds of depression (AOR = 10.94; 95% CI: 4.35, 27.52; *p* < 0.001) than individuals with a normal nutritional status. Similarly, individuals with malnutrition had even greater odds of depression (AOR = 45.61; 95% CI: 13.20, 157.61; *p* < 0.001). Other associated factors that were found to increase the odds of having depression are being female compared with being male (AOR = 2.79; 95% CI: 1.17, 6.63; *p* = 0.02), being retired compared with being employed (AOR = 8.17; 95% CI: 2.18, 30.59; *p* = 0.002) and having sleeping problems compared with being older adult without sleeping problems (AOR = 3.18; 95% CI: 1.43, 7.07; *p* = 0.005). On the other hand, frequent social engagement (5–7 times per week) had a protective effect (AOR = 0.054; 95% CI: 0.009, 0.341; *p* = 0.004) (Table 2).

Moreover, those at risk of malnutrition had greater odds than those with normal nutritional status did (AOR = 11.16; 95% CI: 4.10, 30.41; *p* < 0.001), whereas those with malnutrition had even greater odds of experiencing anxiety (AOR = 17.80; 95% CI: 4.93, 64.32; *p* < 0.001). Other associated factors that were found to increase the odds of anxiety were retirees compared with employed individuals (AOR = 5.28; 95% CI: 1.31, 21.32; *p* = 0.019), smokers (AOR = 4.50; 95% CI: 1.55, 13.11; *p* = 0.006) and ex-smokers (AOR = 8.95; 95% CI: 2.72, 29.51; *p* < 0.001) had higher odds than nonsmokers did, and elderly people with sleeping problems had higher odds than did elderly people without sleeping problems (AOR = 2.99; 95% CI: 1.21, 7.39; *p* = 0.017). On the other hand, engaging in frequent physical activity (5–7 times per week) had a protective effect (AOR = 0.157; 95% CI: 0.041, 0.602; *p* = 0.007), and engaging in less physical activity (2–4 times per week) also had a protective effect against anxiety (AOR = 0.255; 95% CI: 0.079, 0.82; *p* = 0.022) (Table 3).

The odds of stress were also greater in individuals at risk of malnutrition (AOR = 5.03; 95% CI: 1.55, 16.34; *p* = 0.007) and, similarly, in those with malnutrition (AOR = 4.59; 95% CI: 0.82, 25.74), although this association was not statistically significant (*p* = 0.083). Moreover, no other factors were found to be associated with stress (Table 4).

**Table 2:** Multiple logistic regression for factors associated with depression in older adult respondents

Variables	Crude OR (95% CI)	Adj. OR (95% CI)	Wald (df)^a)^	*p*– value^a)^
Nutritional status				
• Normal • At risk of malnutrition • Malnutrition	7.30 (3.43,26.48) 22.1 (8.11,60.21)	1 10.94 (4.35,27.52) 45.61 (13.20,157.61)	42.76 25.83^b)^ 36.46^b)^	< 0.001* < 0.001* < 0.001*
Gender				
• Male • Female	1.70 (0.93,3.21)	1 2.788 (1.173,6.627)	5.38 (1)	0.020
Job Category				
• Employed • Unemployed • Pensioner	2.15 (0.71,6.51) 3.38 (1.11,10.29)	1 2.45 (0.65,9.18) 8.17 (2.18,30.59)	12.176 (2) 1.76 (1)^b)^ 9.72 (1)^b)^	*0.002 0.098^b)^ *0.002^b)^
Sleeping problems				
• No • Yes	2.99 (1.59,5.62)	1 3.18 (1.43,7.07)	8.02 (1)	*0.005
Social activity in a week				
• No activity • Seldom (1 time per week) • Sometimes (2–4 times per week) • Frequent (5–7 times per week)	0.69 (0.30,1.58) 0.34 (0.14,0.81) 0.07 (0.01,0.31)	1 0.865 (0.305, 2.457) 0.352 (0.113,1.101) 0.054 (0.009,0.341)	12.52 (3) 0.074 (1)^b)^ 3.217 (1)^b)^ 9.630 (1)^b)^	*0.006 0.723 0.120 *0.004

Simple logistic regression was initially conducted for all variables, and those with a p value < 0.25 were included in the multiple logistic regression model

*Adj. OR* Adjusted OR, *CI* Confidence interval

^a)^ Likelihood ratio

^b)^Wald test

*p value <0.05

**Table 3:** Multiple logistic regression for factors associated with anxiety in older adult respondents

Variables	Crude OR (95% CI)	Adj. OR (95% CI)	Wald (df)^a)^	*p* -value^a)^
Nutritional status				
• Normal • At risk of malnutrition • Malnutrition	7.57 (3.25,17.63) 11.96 (3.94,36.27)	1 11.16 (4.10,30.41) 17.80 (4.93,64.32)	28.22 22.25^b)^ 19.32^b)^	< 0.001* < 0.001* < 0.001*
Job Category				
• Employed • Unemployed • Pensioner	1.86 (0.52,6.67) 3.56 (1.00,12.61)	1 1.74 (0.42, 7.18) 5.28 (1.31,21.32)	8.232 (2) 0.583 (1)^b)^ 5.469 (1)^b)^	0.016* 0.445 0.019*
Smoking status				
• Non smoker • Smoker • Ex smoker	4.01 (1.71,9.39) 4.98 (1.87,13.29)	1 4.502 (1.55, 13.11) 8.952 (2.72, 29.51)	16.45 (2) 6.009 (1)^b)^ 12.97 (1)^b)^	< 0.001 0.006* < 0.001
Sleeping problems				
• No • Yes	2.49 (1.22,5.10)	1 2.99 (1.21, 7.39)	5.670 (1)	0.017*
Physical Activity in a week				
• No activity • Seldom (1 time per week) • Sometimes (2–4 times per week) • Frequent (5–7 times per week)	0.55 (0.22,1.41) 0.32 (0.12,0.85) 0.19 (0.06,0.59)	1 0.448 (0.14,1.41) 0.255 (0.079, 0.82) 0.157 (0.041,0.602)	8.729 (3) 1.883 (1)^b)^ 5.214 (1)^b)^ 7.280 (1)^b)^	0.033* 0.170 0.022* 0.007*

Simple logistic regression was initially conducted for all variables, and those with a p value < 0.25 were included in the multiple logistic regression model

*Adj. OR* Adjusted OR, *CI* Confidence interval

^a)^ Likelihood ratio

^b)^Wald test

*p value < 0.05

**Table 4:** Multiple logistic regression for factors associated with stress in older adult respondents

Variables	Crude OR (95% CI)	Adj. OR (95% CI)	Wald (df)^a)^	*p* -value^a)^
Nutritional status				
• Normal • At risk of malnutrition • Malnutrition	5.30 (1.70,16.80) 4.76 (0.87,26.05)	1 5.03 (1.55,16.34) 4.59 (0.82,25.74)	7.79 7.23^b^ 3.01^b^	0.020* 0.007* 0.083

## Discussions

In our study, depression (12.7%) emerged as the most common mental health condition experienced by the older adult respondents, followed by anxiety (9.4%) and stress (6.2%). Even though the proportion of these mental health conditions was relatively low, those who presented symptoms had moderate symptoms of depression and anxiety rather than only mild symptoms or transient distress. These results suggest that identification and early intervention are essential to prevent further complications.

The proportion of older population with depression in this study aligned with findings from the National Health Morbidity Survey 2018, which reported an 11% depression rate among older adult in Malaysia [5]. For anxiety, despite the limited number of available studies for comparison, the finding in our study was 9.4%, which was lower than the 22.6% reported among older individuals in rural areas in Perak [7]. Similarly, research in China has shown a greater prevalence of depression in rural areas than in urban areas [25]. These rural–urban differences in the prevalence of depression and anxiety may be influenced by socioeconomic disparities and healthcare access. Given that Kuala Selangor is a mixed urban‒rural district, the lower prevalence observed in this study than in rural areas may reflect better healthcare access, social engagement, and economic opportunities. Additionally, sociocultural factors and community screenings may have contributed to lower depression and anxiety rates by offering stabilizing benefits [26].

Internationally, the prevalence of stress varies widely, with higher rates reported in some regions, such as 40.2% in Iran, where financial insecurity and socioeconomic instability are major contributing factors [27]. This variation may be attributed to the transient nature of stress, unlike depression and anxiety, which tend to persist over time [28]. Given this temporal sensitivity, the lower occurrence (4.2%) in our study with variations in stress prevalence across populations may reflect differences in the timing of assessment rather than stable psychological conditions.

With respect to nutritional status, our screening revealed that nearly 30% of the older individuals were at risk of either malnutrition or malnutrition, which warrants further investigations. These findings are consistent with the findings of the National Health Morbidity Survey 2018, which reported malnutrition and risk rates of 7.3% and 23.5%, respectively, among older adults in Malaysia [29]. Moreover, these conditions are linked to various factors, including age-related physiological changes, restricted access to nutritious food, and the presence of comorbidities [30]. In addition, the prevalence of malnutrition and those at risk among elderly individuals varies across different regions. Our findings are consistent with findings in Singapore, which reported risk rates of malnutrition and malnutrition risk rates of 2.8% and 27.6%, respectively [31]. In contrast, studies in developing countries such as Sri Lanka and India have reported significantly higher malnutrition rates, whereas developed countries such as Spain present a lower prevalence of malnutrition [32, 33]. These findings suggest that regions experiencing greater socioeconomic hardships are well documented contributors to poor nutrition [34]. Furthermore, the relatively lower malnutrition rates observed in our study suggest that our participants may have better access to a stable nutritional environment than those in regions with greater socioeconomic challenges [34]. However, differences in healthcare systems, cultural dietary habits, and availability of social support services across countries may also influence the generalizability of these findings. Therefore, caution is needed when applying these findings beyond the local context.

This study’s novelty lies in identifying a statistical association between nutritional status and mental health problems in elderly individuals, as participants at risk of malnutrition and malnutrition were found to have significantly higher odds of exhibiting depression and anxiety symptoms. Although an association with stress was observed, it was not statistically significant, suggesting a trend that warrants further investigation. This association could be explained by the role of essential nutrients, such as serotonin and dopamine, which are crucial for emotional stability and cognitive function, in supporting neurotransmitter production [11]. In addition, recent findings provide deeper insights into how the gut–brain axis may mediate the effects of nutrition on mental health. The gut microbiota, shaped by diet, produces metabolites such as short-chain fatty acids that regulate immunity, maintain gut barrier function, and influence neurotransmitters related to mood and cognition. Dysbiosis may disrupt these processes, leading to inflammation, impaired neural signalling, and greater risk of mental health disorders [35]. Global studies across diverse regions, including both developed and developing countries such as Bangladesh, Iran, and Norway, similarly reported elevated depression rates among elderly people with malnutrition [12, 36, 37]. However, limited studies are available investigating the effects of malnutrition on anxiety and stress among elderly individuals.

Nonetheless, the very high adjusted odds ratios observed in this study particularly for depression should be interpreted with caution. These strong associations may have been influenced by the relatively small number of participants classified as malnourished, which can result in wide confidence intervals and reduced precision of effect estimates. The wide CIs observed suggest considerable statistical uncertainty, limiting confidence in the exact magnitude of these associations. Moreover, although we adjusted for several confounders, the potential for residual confounding remains. As such, these findings, while statistically significant, should be considered exploratory and interpreted as preliminary evidence that requires validation in larger, more representative samples.

Our study revealed that other factors that were statistically associated with the mental health status of the elderly population. The female sex associated with depression, and increased vulnerability to depression has been attributed to a combination of genetic and biological predispositions, increased healthcare-seeking behaviour, and heightened sensitivity to stressful life events [38, 39]. In contrast, social engagement has a protective effect against depression, with frequent participation in social activities linked to lower depression rates. This finding is supported by a literature review suggesting that social interactions foster mental well-being by reducing loneliness and providing emotional support [40].

Both current smokers and former smokers were found to have higher odds than nonsmokers of having anxiety. This aligns with findings from a longitudinal Irish study on older adults, which reported increased anxiety disorders in smokers due to the impact of nicotine on brain chemistry [41]. In contrast, regular physical activity was identified as a protective factor against anxiety, which is consistent with findings from a large-scale study among community-dwelling elderly individuals in Malaysia [9]. This effect may be attributed to exercise’s ability to boost the production of endorphins, which are neurotransmitters that enhance mood and well-being, thereby reducing anxiety and promoting self-esteem [42].

For both depression and anxiety, retirees in our study, who made up approximately one-third of the sample population, experienced higher odds of depression and anxiety. This finding aligns with analyses of data from six waves of the United States Health and Retirement Study, suggesting that the loss of work roles due to retirement can adversely affect mental health [43]. Sleep problems also emerged as a significant factor for both depression and anxiety, with participants experiencing sleep problems having higher odds. Rapid eye movement (REM) sleep disturbances contribute to depression by altering the balance of monoamine neurotransmitters (serotonin, norepinephrine, and dopamine), which regulate mood and sleep, leading to emotional instability and increased susceptibility to depressive symptoms [44].

Although significant associations were observed, some adjusted odds ratios, particularly for malnourished participants and those with stress, showed very wide confidence intervals (for example, AOR for depression in malnourished individuals: 45.61; 95% CI: 13.20–157.61). This imprecision is likely due to the small number of participants in these subgroups, which may have reduced statistical power and affected the stability of the estimates. These findings should therefore be interpreted with caution. Future studies with larger and more balanced samples are needed to confirm these associations and provide more precise effect estimates.

Nonetheless, despite these statistical limitations, the strength of this study is that we are the only study to examine the associations between nutritional status and all three mental health problems, namely, depression, anxiety, and stress, among older adults. Among the limitations of our study is that the tools we used are screening tools rather than diagnostic tools for identifying mental health problems and malnutrition. In addition, its cross-sectional design limits causal inference, as the direction of association cannot be determined. Malnutrition may contribute to mental health problems, but poor mental health can also lead to reduced appetite and malnutrition, suggesting a possible bidirectional relationship that requires further investigation. Furthermore the focus on Kuala Selangor may limit the generalizability of our findings. In particular, the use of convenience sampling from voluntary health screening attendees introduces a potential selection bias which may have led to an underestimation of the prevalence of both malnutrition and mental health problems. Therefore, caution is needed when applying these findings to broader elderly populations. Subsequently, lifestyle and functional data were based on self-reports without the use of validated instruments, which may introduce measurement bias; however, these variables were collected primarily as potential confounders rather than outcomes of interest. Lastly, certain known confounders such as cognitive status, polypharmacy, and food insecurity were not assessed due to feasibility constraints. These factors may contribute to residual confounding but do not negate the associations observed.

Despite these limitations, the findings of this study have important implications for clinical practice and future research. In geriatric care, integrating mental health assessments can aid in the early identification of elderly individuals at risk of depression, anxiety, and stress, particularly retirees, those with limited social and physical engagement, and individuals with sleep disturbances. Nutritional screening for this high-risk group is essential to enable early intervention through individualized dietary counselling. Specific interventions that could be considered include community-based meal programs, home-delivered nutrition support, and nutrition education workshops tailored for older adults. Social support programs, such as senior activity centers, peer support groups, and community outreach initiatives, may also help improve mental health and reduce isolation in this population. A multidisciplinary approach involving dietitians and healthcare professionals is crucial for comprehensive management, preventing malnutrition-related complications, and enhancing overall well-being.

For future research, longitudinal studies are needed to explore the causal relationship between malnutrition and mental health disorders in elderly individuals. Investigating sociodemographic and lifestyle factors can help refine targeted interventions and improve early detection strategies. Future studies should also assess dietary intake to identify specific nutrient deficiencies and optimize geriatric nutrition guidelines. Additionally, research on the effectiveness of dietary modifications, community-based programs, and personalized interventions can help mitigate nutritional risk and mental health decline. Expanding research to diverse elderly populations, especially those not receiving community screening, will provide a more comprehensive understanding and improve targeted healthcare strategies for vulnerable individuals.

## Conclusion

Although the prevalence of mental health conditions was relatively low in this study, those who exhibited symptoms had moderate symptoms of depression and anxiety rather than only mild symptoms or transient distress, indicating the urgent need for early intervention. Additionally, a significant portion of the elderly have some degree of nutritional issues, implying the importance of assessment and tailored management approaches. Being at risk of malnutrition and being malnourished were found to be strongly associated with depression, anxiety, and stress in the elderly population; however, the direction of this association remains unclear, and reverse causality (for example, depression contributing to poor nutritional intake) cannot be ruled out. Conversely, social engagement and physical activity have a protective effect against mental health problems and should be encouraged in elderly care. In terms of broader implications, these findings support the integration of routine nutritional screening into existing geriatric mental health programs at the primary care level. Policies that incorporate standardized nutrition assessments alongside mental health screening can help identify at-risk individuals earlier and enable timely, multidisciplinary interventions. Strengthening collaboration between family medicine specialists, dietitians, and mental health professionals, as well as expanding community-based initiatives such as senior activity centers and home nutrition support services, may enhance both nutritional status and mental health outcomes among elderly populations.

## Data Availability

The data are provided within the manuscript or supplementary information files.

## Abbreviations

AORs: Adjusted odds ratios
BMI: Body mass index
CI: Confidence interval
DALYs: Disability-Adjusted Life Years
DASS-21: Depression, Anxiety and Stress Scale 21
MNA®-SF: Mini Nutritional Assessment Short Form
SD: Standard deviation
SPSS: Statistical Package for the Social Sciences
